# A Recombinase-Mediated Cassette Exchange Platform for a Triple Independent Inducible Expression System for Human Pluripotent Stem Cells

**DOI:** 10.3390/cells14030184

**Published:** 2025-01-24

**Authors:** Roberto Castro-Gutierrez, Ankita Arora, Katherine F. Vaeth, J. Matthew Taliaferro, Holger A. Russ

**Affiliations:** 1 Diabetes Institute, College of Medicine, University of Florida, Gainesville, FL 32610, USA; 2 Department of Pharmacology and Therapeutics, College of Medicine, University of Florida, Gainesville, FL 32610, USA; 3 Department of Biochemistry and Molecular Genetics, University of Colorado Anschutz Medical Campus, Aurora, CO 80045, USA; ankita.arora@cuanschutz.edu (A.A.); kathrin.vaeth@cuanschutz.edu (K.F.V.); matthew.taliaferro@cuanschutz.edu (J.M.T.); 4 RNA Bioscience Initiative, University of Colorado Anschutz Medical Campus, Aurora, CO 80045, USA

**Keywords:** dual expression system, triple expression system, human pluripotent stem cells, genome engineering, recombinase-mediated cassette exchange (RMCE), tetracycline, degron, cumate, germ layer differentiation, pancreatic, transcription factors, PDX1, NKX6.1

## Abstract

Human pluripotent stem cells (hPSCs) and their differentiated derivatives represent valuable tools for studying development, modeling diseases, and advancing cell therapy. Recent improvements in genome engineering allow for precise modifications of hPSCs, further enhancing their utility in basic and translational research. Here we describe a Recombinase-Mediated Cassette Exchange (RMCE) platform in hPSCs that allows for the highly efficient, rapid, and specific integration of transgenes. The RCME-mediated DNA integration process is nearly 100% efficient, without negatively affecting the pluripotency or karyotypic stability of hPSCs. Taking advantage of this convenient system, we first established a dual inducible expression system based on the Tet-On and Cumate-On systems, allowing for the inducible expression of two transgenes independently. Secondly, we incorporated a Tet-on inducible system, driving the expression of three genes simultaneously. However, two genes also contain independent degron sequences, allowing for precise control over the expression of each gene individually. We demonstrated the utility of these systems in hPSCs, as well as their functionality after differentiation into cells that were representative of the three germ layers. Lastly, we used the triple inducible system to investigate the lineage commitment induced by the pancreatic transcription factors NKX6.1 and PDX1. We found that controlled dual expression, but not individual expression, biases hPSC embryoid body differentiation towards the pancreatic lineage by inducing the expression of the NeuroD program. In sum, we describe a novel genetic engineering platform that allows for the efficient and fast integration of any desired transgene(s) in hPSCs using RMCE. We anticipate that the ability to modulate the expression of three transgenes simultaneously will further accelerate discoveries using stem cell technology.

## 1. Introduction

The genetic engineering of human pluripotent stem cells (hPSCs) represents an invaluable tool for disease modeling and cell replacement therapy [1,2,3,4]. To model and potentially reverse disease, the genetic engineering of hPSCs is a valuable resource. However, the biological and technical challenges of the genetic engineering of hPSC systems make this approach a laborious and time-consuming task. Widely used methods for genome engineering in culture cells include the Clustered Regularly Interspaced Short Palindromic Repeats (CRIPSR-Cas9) system [5] and the Transcription Activator-Like Effector Nucleases (TALEN) system [6]. Despite the fact that these technologies have been widely adopted in many immortalized cell lines and, to some degree, in primary cells, important hurdles are to be considered when translating these techniques to hPSCs. Even though CRISPR has been a revolutionary tool for genetic modifications, off-target effects are a serious concern of this technology [7]. Despite ongoing efforts in reducing the off-target effects of the crRNA [8,9], important challenges still remain, such as PAM sequence dependence and variability in targeting efficiency [10]. TALEN-mediated modifications can provide a great alternative when high specificity is desired. Two TALENs are required, each with a DNA binding domain surrounding the desired target DNA sequence. The TALENs are fused with a split endonuclease, resulting in a specific DNA cleavage only when the TALEN pair come near each other. This mechanism results in a specific genetic modification with low off-target effects. However, due to its cleavage nature, TALEN recombination is often an inefficient process. Alternative methods to facilitate rapid and efficient transgene integration into hPSCs would further advance disease modeling and cell replacement therapy efforts. Recombinase-Mediated Cassette Exchange (RMCE) technology allows for the integration of DNA cassettes through the recognition of specific sequences in the genome [11,12]. However, it was not until the Cre-Lox system was first described in yeast [13] that RMCE began to be widely used in multiple mammalian model systems. By now, RMCE has been widely used in multiple cellular and developmental studies due to its efficiency in in vitro and in vivo models and its spatial and temporal control.

Here, we adapt existing RMCE technology to establish an easy and versatile platform for the efficient integration of transgenes in a site-specific manner into hPSCs. We use this highly efficient system to demonstrate the feasibility of two inducible expression systems in hPSCs. Specifically, our results describe dual and triple inducible expression systems in hPSCs that allow the modulation of up to three transgenes individually. We confirm the accurate and independent expression of three transgenes, including the pancreatic lineage determinants transcription factors PDX1 and NKX6.1 and a reporter gene, Renilla luciferase. Taking advantage of this triple system, we show that the individual expression of PDX1 or NKX6.1 results in the incomplete induction of a pancreatic expression program in an unbiased embryoid body differentiation assay. However, the synergistic activation of both genes results in the induction of the NEUROD expression program and, overall, more complete pancreas expression activation. We anticipate that our findings have important implications for current direct differentiation and disease modeling efforts and will readily be adapted by other researchers in the hPSC field.

## 2. Materials and Methods

### 2.1. hPSC Culture and Maintenance

Undifferentiated human pluripotent stem cells (hPSCs) were maintained on hPSC-qualified Matrigel (Corning #354277) in an mTeSR+ medium (STEMCELL Technologies #05826), as described previously [14].

### 2.2. Genetic Engineering of hPSCs

WT hPSCs or LoxP#10 cells (both from induced pluripotent stem cells from a healthy control, as previously described [14]) were dissociated into single cells using TrypLE by incubation at 37 °C for 8 min. The cells were quenched with an mTESR+ medium, diluted in PBS, and counted using a MoxiGo II cell counter (Orflow). Then, 2 × 10^6^ cells were transferred into microcentrifuge tubes, spun down, and washed twice with PBS. The cells were then prepared for the nucleofection of TALEN-mediated knock-in (KI) (i) or Cre-mediated RCME (ii). The cells were nucleofected in a P3 buffer following the Amaxa P3 Primary cell 4D-Nucleofector kit protocol (V4XP-3024) using program CB-150. Next, (i) 0.5 μg of AAVS1-TALEN-L and AAVS1-TALEN-R (gift from Dr. Danwei Huangfu, (Addgene plasmid #59025), as well as 2 μg of targeting plasmid, were used for the nucleofection of the hPSCs. The nucleofected hPSCs were plated in 10 cm plates with 10 μM of ROCK inhibitor, 1× CloneR (STEMCELL # 05888), and 1 μM of SCR7 (Excess Bioscience #M60082). Forty-eight h after plating, Blasticidin selection (10 μg/mL) was performed for 10 days. The surviving clonal colonies were picked and expanded for characterization. (ii) For RMCE, 7 × 10^6^ cells were electroporated with a constitutive Cre plasmid and a RIPE cassette using a BioRad GenePulser electroporation system using an exponential decay with 250 V and 500 μF settings, as described previously [15]. The electroporated cells were plated in 10 cm plates with 10 μM of ROCK inhibitor and 1× CloneR. Forty-eight h after plating, puromycin selection (0.5 μg/mL) was performed for forty-eight h. The clonal colonies were picked after ~10 days and expanded for further characterization.

### 2.3. hPSC Differentiation into Different Germ Layers

The confluent hPSC cultures were dissociated into single-cell suspensions by incubation with TrypLE (Gibco #12-604-021) for 8 min at 37 °C. The detached cells were quenched with an mTESR+ medium and diluted in PBS. The cells were counted using a MoxiGo II cell counter (Orflow). Then, 6 × 10^5^ cells were seeded into multiple 24-well plates in an mTeSR+ medium supplemented with 10 μM of ROCK inhibitor (Y-27632, R&D Systems #1254-50). Twenty-four h later, the medium was changed to mTESR+ without ROCKi. When the cells were confluent, or 1–2 days later, the differentiation into each of the three germ layers was performed as follows.

### 2.4. Endoderm Differentiations Were Carried Out as Previously Described [16]

Briefly, the confluent hPSCs were washed once with PBS and the differentiation was started by adding 500 μL of medium AC (RPMI containing 0.2% FBS, 1:5000 ITS (Gibco #41400-045), 100 ng/mL Activin A (R&D Systems #338-AC-01M), and 0.3 μM CHIR99021 (STEMCELL Technologies #72054)). Next, 24 and 48 h later, the medium was changed to 500 μL of medium A (RPMI containing 0.2% FBS, 1:2000 ITS, and 100 ng/mL Activin A). On day 3, the hPSC-derived definitive endodermal cells were collected for downstream analyses.

### 2.5. Ectoderm Differentiations Were Carried Out as Previously Described [17]

Briefly, confluent hPSCs were washed once with PBS and differentiation was started by adding 500 μL of medium MN (KO DMEM/F12 (Gibco)), containing 1× N2-A Supplement (Stem Cell Technologies), 1× SM1 (Stem Cell Technologies), 1× Glutamax (Gibco), 50 mM of ascorbic acid (Sigma), 1 μM of Compound C, 1 μM of CHIR, 10 μM of Rock Inhibitor, and 1 μM SB431542. The medium was changed every 24 h for a total of 6 days, after which, the hPSC-derived ectodermal cells were collected for downstream analyses.

### 2.6. Mesoderm Differentiations Were Carried Out as Recently Described [18]

Briefly, the confluent hPSCs were washed once with PBS, and the differentiation was started by adding 500 μL of medium ABCFP (Base medium: KO DMEM/F12 (Gibco), containing 1× N2-A Supplement (Stem Cell Technologies), 1× NEAA (Gibco), 1× Glutamax (Gibco), 1× P/S (Gibco), 1× Sodium Pyruvate (Gibco), and 15 mM Hepes (Gibco) with the following factors: 30 ng/mL ActivinA, 40 ng/mL BMP4, 6 μM CHIR, 20 ng/mL FGF2, and 100 nM PIK90). Next, 24 h later, the medium was changed to 500 μL of ABC (Base medium containing 1 μM A8301, 30 ng/mL BMP4, and 1 μM C59. Thereafter, the cells were fed daily with 500 μL of medium ABCFR (base medium with 1 μM A8301, 30 ng/mL BMP4, 1 μM C59, 20 ng/mL FGF2, and 6 μM TTNPB). On day 5, the hPSC-derived mesodermal cells were collected for downstream analyses.

### 2.7. EB Differentiation from hPSCs

The hPSCs were dissociated into single cells and counted using the Countess cell counter. Homogeneous hPSC clusters with 1000 cells/cluster were generated by plating ~5.9 × 10^6^ cells into a 6-well 400 Agrewell plate (STEMCELL) and spun down. The clusters were cultured in this plate with an mTeSR plus medium supplemented with 10 μM of ROCKi. After 24 h, the medium was changed and supplemented with the different treatments for 48 h: DOX, DOX + TMP + dTAG (DTG), and DOX + TMP (DT). After this incubation period, the hPSC clusters were then transferred into a suspension 6-well plate with an EB differentiation medium (DMEM F12 supplemented with 10% FBS) and cultured for 7–8 days in a shaker plate (100 rpms) at 37 °C, 5% CO_2_, and 95% humidity.

### 2.8. Immunofluorescence Staining

The hPSCs and the differentiated derivates were dissociated into single-cell suspensions. The cells were washed with PBS and incubated with TrypLE at 37 °C for 8 min to create a single-cell suspension. The cells were then quenched with 2% FBS in PBS and fixated with 4% paraformaldehyde. Antibody dilutions were prepared in PBS with 0.4% Tween-20. Images were acquired using a confocal microscope (Carl Zeiss LSM 800).

### 2.9. Flow Cytometry

The hPSCs and the differentiated derivates were collected and dissociated, as outlined above. Single cells were filtered through a cell strainer into FACS 5 mL tubes, washed, and resuspended in a FACS buffer (PBS with 0.1% FBS and 0.1% Tween-20). The cells were then run in a CYTEK Aurora flow cytometer. The analysis and graphs were carried out using FloJo software v10.6.2.

### 2.10. RT-qPCR

Total RNA was isolated using a micro-RNeasy kit (Qiagen #74104) and reverse transcribed using the iSCRIPT cDNA kit (BioRad #1708891), as per the manufacturer’s instructions. A qPCR analysis was performed on the BioRad CFX96 Real Time System.

## 3. Results

### 3.1. The Establishment of a RMCE Platform in hPSCs

**Figure 1:**
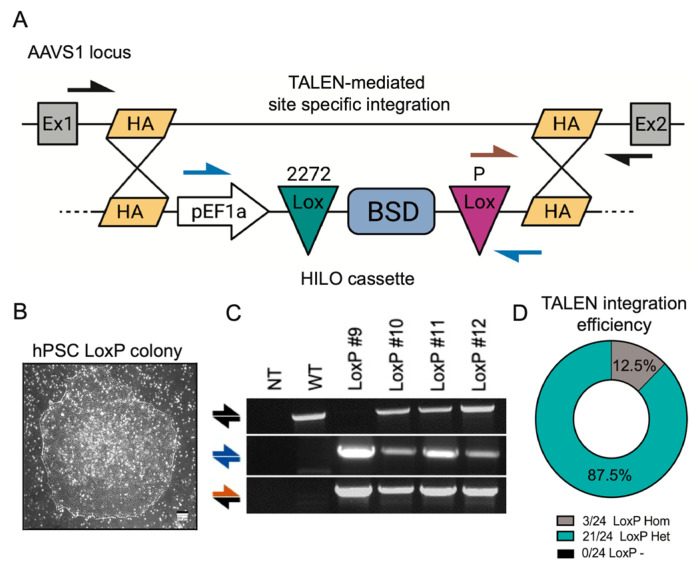
**The TALEN-mediated integration of a HILO cassette in hiPSCs**. (**A**) A schematic diagram of the TALEN-mediated integration of the HILO cassette into the AAVS1 locus of a hiPSC. Arrows indicate primer binding locations. (**B**) Pluripotent colony image after HILO integration and 10 days of selection with 10 μg/mL BSD. (**C**) The genomic DNA PCR analysis for the amplification of the WT sequence (top row), HILO cassette sequence (middle row), and AAVS1 site-specific integration of the HILO cassette (bottom row). (**D**) Pie chart showing efficiency of HILO cassette integration into 24 different hPSC colonies.

In this study, we employed an in-house-generated, human-induced pluripotent stem cell (hPSC). The generation of the hPSCs was accomplished by reprogramming CD34+ hematopoietic progenitor cells using none-integrative Sendia viruses encoding four reprogramming factors (OCT4, SOX2, KLF4, and c-MYC) from a healthy individual. To determine the feasibility of RMCE technology in hPSCs, we integrated the high-efficiency and low-background (HILO) cassette into the AAVS1 locus (also known as PPP1R12C) using TALEN technology [19]. The HILO-=targeting cassette consisted of a EF1α promoter sequence driving the constitutive expression of a Blasticidin resistance gene (BSD) flanked by lox2272 and loxP sites (Figure 1A). The construct also contained homology arms corresponding to exon 1 and exon 2 of the endogenous AAVS1 loci, allowing for site-specific integration via TALEN (Figure 1A). Of note, the disruption of the AAVS1 gene in hPSCs does not result in an altered phenotype or a loss of pluripotency [20]. The integration of the HILO cassette was achieved by the nucleofection of the HILO cassette and two TALEN plasmids in hPSCs, followed by BSD selection. To improve cell survival and promote homology-directed DNA repair, the inhibition of Rho-associated, coiled-coil-containing protein kinase (ROCK) and the inhibition of non-homologous end-joining (NHEJ) pathways were facilitated by adding the small molecules Y-27632 and SCR7, respectively [21]. After 10 days of BSD selection (10 μg/mL), BDS-resistant and clonal pluripotent healthy colonies emerged and were manually picked and expanded for the downstream analysis (Figure 1B). A genomic DNA PCR analysis was used to determine the site-specific integration of the HILO cassette in different colonies. The presence of the cassette was confirmed by using primers amplifying an internal cassette region (blue arrows) (Figure 1A,C). Combining primers, which bind outside the homology (black arrows) and inside the cassette (brown arrows), were used to confirm the site-specific integration of the cassette into the AAVS1 locus (Figure 1A,C). The screening of 24 colonies revealed a high efficiency (87.5%) for the heterozygotic, site-specific integration of the HILO cassette, while homozygotic integration was only observed in 12.5% of the clones. Additionally, none of the clonal colonies analyzed were found to be negative for HILO cassette integration, confirming effective BSD selection (Figure 1D). To avoid unwanted recombination between two HILO copies, present in homozygotic clones, one of the identified heterozygous clones (hereafter referred to as LoxP#10) was randomly chosen for further experimentation. The detailed phenotypical analysis of LoxP#10 cells showed the uniform protein expression of the pluripotent markers SOX2 and TRA160 and a normal karyotype (Supplementary Figure S1A). Altogether, these data demonstrate the efficient integration of the HILO cassette into an hPSC line without affecting pluripotency marker expression.

### 3.2. The Efficient Generation of a Dual Inducible System in hPSCs by RMCE

**Figure 2:**
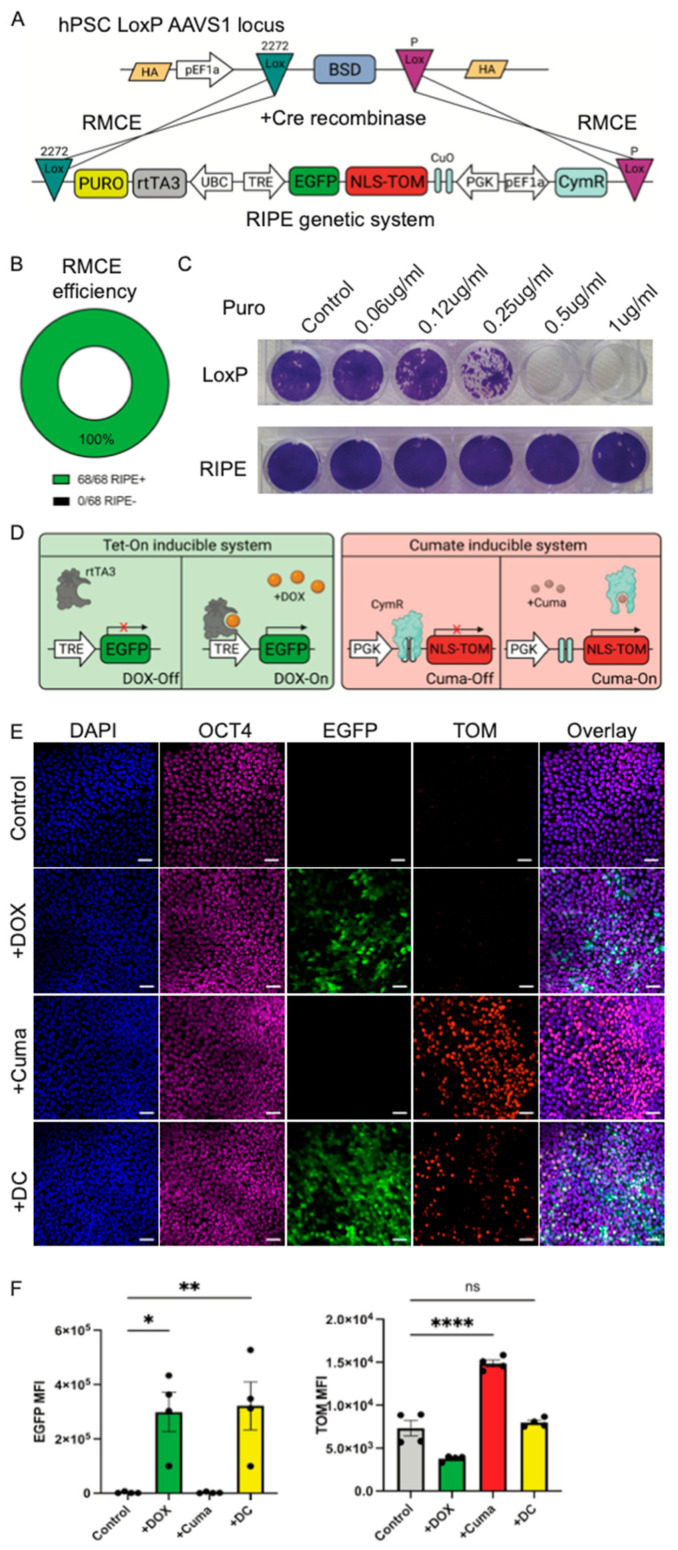
**The RMCE-mediated integration of a functional dual inducible gene expression system.** (**A**) A schematic diagram for the Cre-mediated recombination of the RIPE cassette into the HILO cassette in the AAVS1 of a LoxP#10 hiPSC. (**B**) A pie chart showing the efficiency of the RMCE recombination of the RIPE cassette into the HILO system in 68 different colonies. Efficiency of reconditioning was measured by detecting GFP+ colonies after addition of DOX. (**C**) Crystal violet staining of surviving LoxP#10 and RIPE#2 hiPSCs treated with increasing concentrations of puromycin. (**D**) A schematic representation of the Tet-On (left) and the Cuma-On inducible systems. The addition of DOX allows for the binding of the trans activator rtTA3 to the tetracycline responsive element (TRE) promoter, inducing the expression of EGFP. The addition of Cuma releases the repressor CymR from the CuO binding sites, inducing the expression of TOM from a PGK constitutive promoter. (**E**) Immunofluorescence images of hiPSC RIPE#2 cells treated with DOX, Cuma, or both. DAPI was used to visualize cell nuclei. OCT4 pluripotent marker is in magenta, EGFP in green, and TOM in red. Scale bar represents 50 μM. (**F**) Flow cytometric quantification of EGFP (left panel) and TOM (right panel) mean fluorescence intensities of induced hiPSC RIPE#2. Each dot represents an independent experiment. Statistical analysis includes One-Way Anova. * = 0.05, ** = 0.01, **** = 0.001.

To test the functionality of RMCE in LoxP#10 hPSCs, we integrated a dual inducible expression system (RIPE). The RIPE cassette contains all the components required to facilitate the inducible gene expression of two transgenes and includes a promoter-less puromycin resistance. The directional and site-specific integration of the RIPE cassette into the HILO platform via Cre recombination results in an in-frame integration. Specifically, the fact that the EF1α promoter is outside of the lox2272 site and that the RIPE system contains a promoter-less puromycin resistance gene allows for the convenient identification of site-specific integration (Figure 2A). LoxP#10 hPSCs were nucleofected with the RIPE plasmid and a second plasmid that constitutively expressed Cre recombinase. Forty-eight h post-nucleofection, the cells were selected with puromycin (0.5 μg/mL). Colonies emerged and were manually picked and expanded for downstream testing. The clonal analysis of a total of 68 puromycin-resistant colonies revealed a 100% recombination efficiency (Figure 2B). RIPE clone #2 was randomly chosen for further experimentation (hereafter referred to as RIPE#2). We exposed LoxP#10 or RIPE#2 to a puromycin titration curve. The LoxP#10 parental line, containing the Blasticidin but not the puromycin resistance gene, started showing obvious cell death after 48 h at 0.25 μg/mL puromycin and complete killing at 0.5 μg/mL. The RIPE#2 cells, containing the puromycin resistance, survived even in the presence of 1 μg/mL puromycin (Figure 2D). These results provide further evidence for successful RMCE, resulting in functional puromycin resistance gene expression. Of note, the further analysis showed that RIPE#2 cells express the pluripotent markers SOX2 and TRA160 and exhibit a normal karyotype (Supplementary Figure S2B). Next, we sought to evaluate the functionality of the dual inducible system integrated into RIPE#2 hPSCs. Inducible expression in this system is achieved via the addition of Doxycycline (DOX) and/or Cumate (Cuma). Constitutive promoter sequences within the RIPE construct drive the ubiquitous expression of rtTA3 and, bicistronically, CymR (Figure 2C). The addition of Doxycycline (or tetracycline) facilitates the binding of the rtTA3 protein to tetracycline responsive elements (TREs), resulting in the expression of an enhanced green fluorescence protein (EGFP) (Figure 2D). CymR, unlike rtTA3, is bound to an operator site, CuO, located between a constitutive promoter (PGK) and a dTomato fluorescence protein (TOM) sequence containing a nuclear localization signal (NLS), thereby preventing its expression in the absence of Cuma. Cuma binds to the CymR, releasing it from its operator site, CuO. This results in the induction of TOM expression, driven by the constitutive PGK promoter (Figure 2D). Indeed, the addition of DOX to RIPE#2 cells resulted in the robust cytoplasmic expression of EGFP, while Cuma addition induced moderate TOM expression and localization to the nucleus (Figure 2E). The combined addition of DOX and Cuma (DC) resulted in the robust induction of EGFP, while TOM expression was lower, yet still visibly induced. Importantly, the expression of transgenes did not affect the pluripotency of the cells, as verified by protein staining for the pluripotency marker OCT4 (Figure 2E). Flow cytometric quantification showed the significant expression of EGFP after the DOX or DC addition, confirming the microscopic analysis (Figure 2F). No EGFP expression was detected in the absence of DOX. The quantification of TOM fluorescence after Cuma addition alone showed significantly increased expression. However, even in the absence of Cuma, low levels of TOM expression were detected, indicating some degree of leakiness in the CymR system. The combined addition of DC resulted in readily identifiable TOM protein in some, but not all, the cell nuclei, using microscopy (Figure 2E). TOM expression did not reach significance when the fluorescence intensity of the total cells was quantified via flow, verifying the microscopic analysis (Figure 2F). These data demonstrate the efficient integration of a dual inducible expression system into hPSCs using RCME.

### 3.3. RIPE System Remains Functional After the Differentiation of the hPSCs into the Cells of the Three Germ Layers

**Figure 3:**
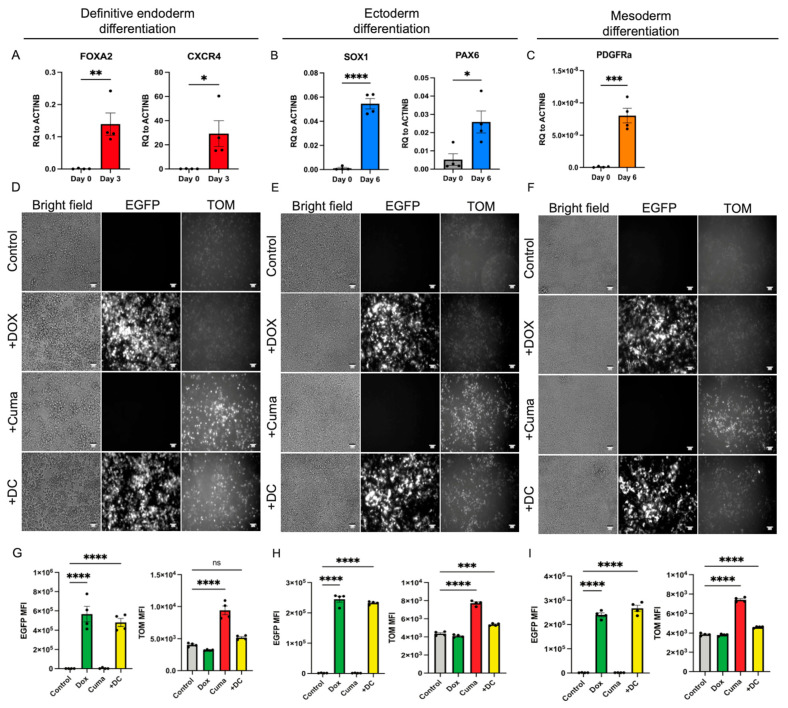
**RIPE#2 system remains functional after differentiation of hiPSCs into germ layers.** qPCR analysis of endoderm (**A**), ectoderm (**B**), and mesoderm (**C**) genes on day 0 and day 3 or 6 after differentiation of hiPSCs. Bright field and epifluorescence images of differentiated hiPSC RIPE#2 cells into endoderm (**D**), ectoderm (**E**), and mesoderm (**F**) after addition of DOX, Cuma, or both. Flow cytometric quantification of EGFP and TOM mean fluorescence intensities of endoderm- (**G**), ectoderm- (**H**), and mesoderm-differentiated (**I**) RIPE#2 cells. Each dot represents an independent experiment. Statistical analysis includes One-Way Anova. * = 0.05, ** = 0.01, *** = 0.005, **** = 0.001.

To further test the function of the dual RIPE system in the cells derived from the hPSCs, we differentiated the RIPE#2 cells into derivatives of the three germ layers using published protocols [16,17,18]. The RIPE#2 cells were dissociated into single cells and plated onto 24-well plates. Next, 48 h after plating, differentiation was induced, as described in the Section 2. Endoderm differentiation was performed for 3 days while ectoderm and mesoderm differentiation were carried out for 6 days. Effective direct differentiation was evaluated by a qPCR analysis for key lineage markers: FOXA2 and CXCR4 (endoderm), SOX1 and PAX6 (ectoderm), and PDGFRα (mesoderm), respectively (Figure 3A–C). To induce RIPE transgene expression, individual cultures were exposed to DOX or Cuma, alone or in combination (DC), for the last 48 h of the differentiation. Untreated cells served as controls. Cytoplasmic EGFP expression and nuclear TOM expression were detected by epifluorescence microscopy in the endoderm-, ectoderm-, and mesoderm-differentiated RIPE#2 cells after the addition of DOX, Cuma, or DC (Figure 3D–F). A flow cytometric quantification verified significant increases in the mean fluorescence intensity (MFI) in all the treated conditions, except for the induction of TOM in definitive endoderm cells after the DC treatment. However, individual cells that were positive for nuclear TOM could be identified using microscopy in the DC-treated endoderm cells, similarly to the experiments conducted using hPSCs (Figure 2E). Overall, these data show that the RIPE system remains functional after differentiation into cells of the three germ layers. Furthermore, these results provide further evidence that RIPE#2 hPSCs retain their pluripotent potential. Taken together, we present here detailed information on the generation of an RCME system in hPSCs and further use this approach to establish a dual inducible system that is functional in hPSCs and differentiated derivatives. We anticipate that the ability to modulate the expression of two transgenes (or other regulator sequences, e.g., shRNAs) simultaneously will further accelerate ongoing research efforts.

### 3.4. The Integration of an Improved, Degron-Containing Triple Inducible System (pTriple) into LoxP10 hPSCs Using RMCE

**Figure 4:**
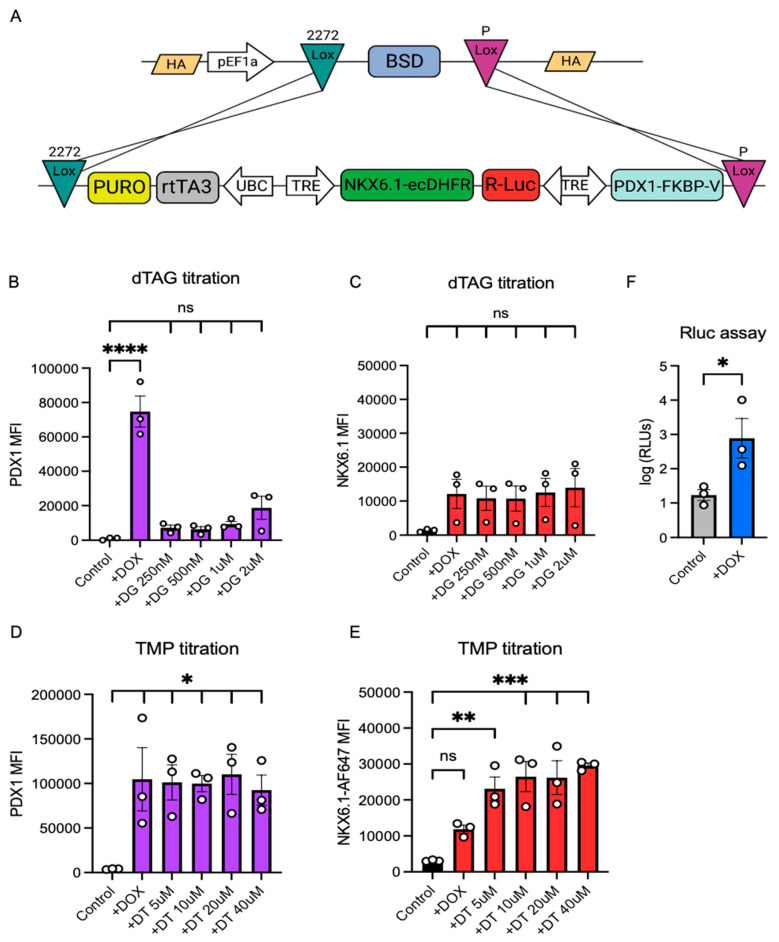
**The integration of a functional triple inducible system (pTriple) into LoxP10 hPSCs using RMCE.** (**A**) A schematic diagram of the RMCE-mediated integration of a triple inducible genetic system into LoxP10 hPSCs using the HILO system. The integrated HILO cassette contains two different LoxP sites (Lox 2272 and LoxP) that match the LoxP sites on the LoxP10 AAVS1 locus. Immediately after Lox2272 is a puromycin resistance gene that allows for site-specific integration after selection. In between the LoxP sites, a Tet-On inducible system controlling the expression of PDX1, NKX6.1, and Renilla luciferase genes is incorporated. Trans activator rtTA3 is controlled by the expression of the constitutive UBC promoter. The PDX1 gene is attached to the FKVP^F36V^ degron on the C-terminus. NKX6.1 is attached to the ecDHFR degron system on the C-terminus. Mean fluorescent intensity of PDX1 (**B**) and NKX6.1 (**C**) expression in hPSC pTriple cells after DOX addition and titration of dTAG (DG) from 250 nM to 2 μM. Mean fluorescent intensity of PDX1 (**D**) and NKX6.1 (**E**) expression in hPSC pTriple cells after DOX addition and titration of TMP (DT) from 5 μM to 40 μM. Each dot represents an independent experiment. Statistical analysis includes One-Way Anova. (**F**) Relative light units (RLUs) in logarithmic scale, representing Renilla luciferase activity in pTriple hPSCs after DOX addition. Each dot represents an independent experiment. The statistical analysis used was a Student’s *T*-test. * = 0.05, ** = 0.01, *** = 0.005, **** = 0.001.

Next, we sought to advance the dual RIPE system to be able to more precisely control individual transgene expression and extend the existing capacity, which was restricted to two transgenes. We generated a plasmid containing a Tet promoter that controlled the expression of three genes: PDX1, NKX6.1, and Renilla luciferase. To control the individual expression of either the PDX1 or NKX6.1 genes, we inserted the FKVP^F36V^ or ecDHFR degron systems into the C-terminus of PDX1 and NKX6.1, respectively (Figure 4A). Both degron systems work independently from each other and allow for the rapid degradation of proteins containing the FKVP^F36V^ domain by adding the degradation tag (dTAG) or to stop the continuous degradation of proteins containing the ecDHFR domain by adding the antibiotic trimethoprim (TMP). We refer to this new triple inducible cassette as pTriple. We integrated the pTriple system into the LoxP10 cell line using RMCE, as described above. To test whether the expression of the PDX1 containing the FKVP^F36V^ domain can be controlled by dTAG, we performed a dTAG titration analysis of the hPSCs after DOX addition. The DOX addition resulted in the strong upregulation of the PDX1 protein, while supplementation with dTAG strongly prevented PDX1 expression, even at low doses (250 nM) (Figure 4B). The addition of DOX and dTAG did not affect the expression of the NKX6.1 protein significantly, indicating the effective degradation of the protein in the absence of TMP (Figure 4C). Next, we investigated whether the NKX6.1 protein could be stabilized in the presence of DOX and TMP. We observed that addition of TMP to the hPSCs after the DOX addition resulted in a significantly increased NKX6.1 expression stability in a dose-dependent manner (Figure 4E). The addition of TMP, even at a high concentration (40 μM), did not impact the expression of the PDX1 containing the FKVP^F36V^ domain (Figure 4D). Lastly, we performed a luciferase assay on the pTriple hPSCs and found that after the addition of DOX, Renilla luciferase activity was significantly upregulated. Taken together, these data show the feasibility of controlling the precise induction of three different individual genes in hPSCs. We believe this tool can be especially useful for the investigation of the temporal expression of multiple transcription factors during hPSC differentiation.

### 3.5. The Dual, but Not Single, Induction of PDX1 and NKX6.1 Protein Expression Is Sufficient to Induce a Pancreatic Expression Program Including NEUROD

**Figure 5:**
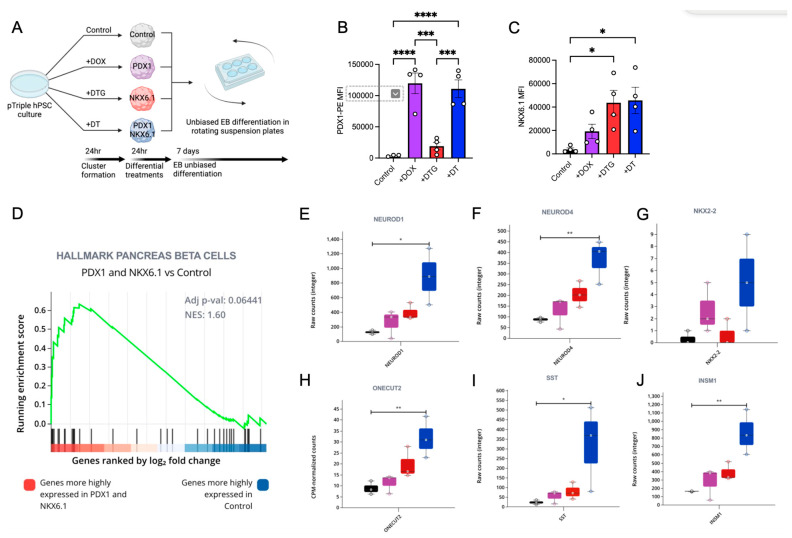
A multiple and independent gene inducible system reveals that PDX1 and NKX6.1 are sufficient to upregulate the NeuroD program during unbiased hPSC differentiation. (**A**) A schematic diagram of the unbiased hPSC differentiation protocol and the different types of molecule combinations for independent gene expression. DOX + TMP + dTAG (+DTG) and DOX + TMP (+DT). A bar graph summarizing the results from the intracellular flow cytometry analysis of PDX1 MFI (**B**) and NKX6.1 MFI (**C**) in hPSC clusters after differential treatment with different molecules and before inducing unbiased differentiation. (**D**) The GeneSet enrichment analysis of the RNA-seq between PDX1 and NKX6.1 conditions (+DT) and the control, showing an enrichment of the hallmark pancreatic beta cells being highly expressed in the +DT condition. The raw counts of single genes after the differential gene expression analysis between all the different treatments, showing the expression of (**E**) NEUROD1, (**F**) NEUROD4, (**G**) NKX2.2, (**H**) ONECUT2, (**I**) STT, and (**J**) INSM1. Each dot represents an independent experiment. Statistical analysis includes One-Way Anova with multiple comparisons against control condition. * = 0.05, ** = 0.01, *** = 0.005, **** = 0.001.

To investigate the effects of the individual or combined activation of PDX1 and/or NKX6.1 expression, we generated hPSC 3D clusters using the pTriple hPSC line described above. We then treated the pTriple hPSC clusters with DOX alone to induce PDX1, with DOX + TMP + dTAG (DTG) to induce NKX6.1, or with DOX + TMP (DT) to induce both PDX1 and NKX6.1 transgenes expression (Figure 5A). We validated the effective induction of PDX1 and/or NKX6.1 protein expression via flow cytometry base quantification (Figure 5B,C). Confirming our previous results, the DOX and DT treatment strongly upregulated PDX1, while the addition of dTAG in DTG treatment significantly reduced PDX1 expression. The addition of TMP alone in the DT treatment did not significantly affect PDX1 expression. All the treatments with DOX upregulated NKX6.1; however, significant upregulation was only observed when TMP was added, namely in the DTG and DT treatments (Figure 5C). After the cluster formation and treatments, the hPSC clusters were exposed to unbiased embryoid body (EB) differentiation for 7 days (Figure 5A). At the end of the differentiation, an RNA-seq analysis was performed on the clusters from all the conditions. A principal component analysis (PCA) showed great variability between all the samples, as expected of EB-based unbiased differentiation approaches (Supplementary Figure S2). Despite this variability, we were able to detect an enrichment score for the hallmarks of pancreatic beta cells with an NES of 1.6 when comparing the control group with the *DT* group expressing high levels of both PDX1 and NKX6.1 (Figure 5D). This result was expected since these genes are highly expressed on pancreatic progenitors [22,23]. Additionally, a further differential gene expression analysis showed the strong upregulation of the NeuroD program in all the conditions, but this was most pronounced in the PDX1- and NKX6.1-primed EB differentiation. Specifically, the NeuroD1 and NeuroD4 genes were significantly upregulated (Figure 5E,F). These data suggest that the PDX1 and NKX6.1 genes are sufficient to induce the expression of the NeuroD program and that they exhibit a synergistic effect. Moreover, by comparing the gene expression analysis of the DOX and the DTG treatments we were able to determine the individualistic role of PDX1 and NKX6.1. We found that PDX1 overexpression in the hPSC state, followed by unbiased EB differentiation, is sufficient to upregulate the endocrine marker NKX2.2 (Figure 5G), while the overexpression of NKX6.1 was sufficient to upregulate ONECUT2 expression (Figure 5H). A synergistic effect of PDX1 and NKX6.1 overexpression was also observed in the expression of these two genes. Interestingly, we observed a strong synergistic effect on the upregulation of the hormone SST (Figure 5I) and the transcription factor INSM1 (Figure 5J). INSM1 has been previously reported as a direct target of Neurog3-initiated pancreatic endocrine differentiation, further supporting the notion that PDX1 and NKX6.1 act synergistically to induce a pancreatic phenotype [24]. Altogether, these data show that the upregulation of NKX6.1 and PDX1 prior to unbiased EB differentiation can bias the lineage towards pancreatic endoderm- and SST-producing cells. Moreover, by utilizing a pTriple inducible system, we were able to dissect the individualistic and synergetic roles of PDX1 and NKX6.1 during hPSC differentiation. Finally, these data show the functionality and biological relevance of a triple inducible system in hPSCs that can control the expression of multiple genes in the same hPSC line and allows for the specific dissection of gene interactions during a model of human development. We anticipate that the ability to modulate three transgenes simultaneously will be readily adapted by the wider stem cell research community.

## 4. Discussion

The genetic engineering of hPSCs represents a powerful tool for modeling human development, drug discovery, cell replacement therapy, disease modeling, and personalized medicine. Despite recent advances, the genomic engineering of hPSCs remains a complicated and laborious task. Thus, alternative approaches to facilitate accurate, fast, and easy gene modification are highly sought after. In this study, we adopted an RCME strategy based on the widely used Cre-Lox system in hPSCs. Our strategy results in the rapid and efficient transgene integration of any desired DNA sequence without affecting the pluripotency state of the modified cells. We achieved this by first generating a parental RMCE-compatible cell line by site-specifically integrating a high-efficiency and low-background (HILO) cassette into the AAVS1 loci using TALEN technology. The HILO cassette contains an EF1α promoter, driving the constitutive expression of a floxed, BSD-resistant gene, allowing for the convenient selection of targeted clones using antibiotics. The two Lox sites flanking BSD are unique, thus allowing the directional integration of any DNA sequence that is also flanked by both Lox sites in the presence of Cre expression. By utilizing a promoter-less, puromycin-resistant gene in the RMCE-compatible, RIPE-targeting constructs, we ensure selection by puromycin in clones with site-specific integration. Indeed, this approach results in the highly efficient integration of transgenes, mediated by RMCE. We observed a 100% efficiency in transgene integration after 48 h of puromycin selection. Of note, we have used the RIPE system for multiple other transgenes in our lab and have observed similar efficiencies. This further supports our notion that RIPE represents a useful pluripotent stem cell tool. The high efficiency of RMCE suggests that picking and screening colonies can be kept to a minimum or omitted altogether.

Previously, developmental studies used the Cre-Lox technology for genetic modifications in animal models. Here, we created an RMCE-compatible hPSC line, representing a valuable tool for translating non-human research approaches into a human model system. We employed this system to show the feasibility of a dual inducible expression system in hPSCs using RMCE. This technology can be further applied for the integration of alternative transgenes. In addition to transgene expression, inducible hairpin RNA interference (shRNAi) expression to knock down the gene expression of interest can be conveniently implemented using our system [19]. To subject our genetic engineering platform to modeling human development, the RIPE#2 cells were differentiated into the three germ layers. The gene expression analysis verified marker gene expression for endoderm, mesoderm, and ectoderm differentiation. Importantly, the dual inducible expression system remained functional after the differentiation, indicating its potential for transgene activation after the differentiation of target cell types from any of the three germ layers. Specifically, the Tet-On and Cuma-On systems remained functional after the differentiation, with the Cuma-On system leaking some basal TOM expression. The dual induction of both EGFP and TOM using DOX and Cuma resulted in the strong expression of EGFP but the minor expression of TOM. This suggests that the bicistronically expressed EGFP might be interfering with the Cuma system. Additional modifications to the design can be used to overcome this issue.

To improve the leakiness of the transgene expression, we designed a triple expression system based on the Tet-On inducible system and combined it with the addition of degron domains at the C-terminus of the transgenes (pTriple system). This novel system was cloned into the HILO-targeting cassette and integrated into the genome of the LoxP10 hPSCs using RMCE, again with 100% efficiency. The analysis of the functionality of the pTriple system verified the improved functionality of the induction of three transgenes: PDX1, NKX6.1, and Renilla luciferase. The activation of the FKBP degron domain in PDX1 showed the efficient degradation of the PDX1 protein. The ecDHFR domain acts by constantly degrading the NKX6.1 transgene via proteasome. Upon the addition of TMP, this pathway is disrupted, and the protein stabilizes. However, when NKX6.1-ecDHFR is induced by DOX, a low level of NKX6.1 protein can be detected without the addition of TMP. However, we did not detect any obvious biological effects of this transgene’s leakiness during the course of this study.

To demonstrate the utility of the pTriple hPSCs system, we biased the EB differentiation towards the pancreatic lineage by inducing NKX6.1 and PDX1 protein expression temporarily. The results from these experiments showed that PDX1 and NKX6.1 have a synergistic effect in the differentiation of EBs towards a pancreatic lineage. The GeneSet enrichment analysis showed a trend towards pancreatic beta cell genset enrichment when PDX1 and NKX6.1 were co-overexpressed during the EB differentiation. The expression of pancreatic lineage genes, such as NeuroD1, NeuroD4, NKX2.2, ONECUT2, SST, and INSM1, was upregulated when PDX1 and NKX6.1 were overexpressed. Differential effects, albeit more modest, were also observed when the genes were individually expressed. No significant upregulation of the islet hormones INS or GCG was detected; however, SST was significantly upregulated. This might point towards the effect or the inadequate activation of premature endocrine differentiation in cells that were not properly specified. We previously reported that improper endocrine differentiation results in unwanted hormone expression, which might explain the biased differentiation towards SST-producing cells observed here. Overall, this independent multiple-gene inducible system in hPSCs allowed us to investigate the role of PDX1 and NKX6.1 expression and show the specific synergistic upregulation of the NeuroD program in hPSCs, without additional direct differentiation clues being provided. These results provide evidence for the applicability and usefulness of the pTriple system in investigating the directed modulation of gene regulatory networks in combination with up to three transgenes using differentiation protocols for up to 6 days. Future studies will aim to examine the pTRIPLE system in longer direct differentiation protocols, for example, differentiation into functional human beta cells.

## 5. Conclusions

Conclusively, Cre-Lox technology-mediated RCME technologies, as described here, have considerable advantages over CRISPR-Cas9 and TALEN systems. (I) Cre-Lox technology only requires a straightforward, efficient recombination event, mediated by the Cre enzyme. This results in highly efficient and specific recombination events, reducing the time and the cost of genome engineering. (II) The Cre-Lox system is highly specific. Cre-mediated recombination requires Lox sequences in the genome. Innately, hPSCs do not contain Lox sequences, directing the RCME event to the modified locus: in this study, the AAVS1 locus. (III) The Cre-Lox system here has been optimized for the rapid integration of transgenes. Due to the high efficiency of the RMCE shown here, no colony picking, amplification, or screening is effectively required. Furthermore, no impacts on pluripotency or detrimental karyotypic instabilities were observed. We believe that our Cre-Lox system represents a practicable tool for the integration of multiple transgenes into the AAVS1 locus of hPSCs and can be further exploited to recapitulate developmental and disease modeling studies in a relevant human development model system.

## Data Availability

GEO accession number GSE283985.

## Supplementary Materials

The following supporting information can be downloaded at https://www.mdpi.com/article/10.3390/cells14030184/s1, Figure S1: Characterization of LoxP#10 and RIPE#2 hiPSCs. Flow cytometric plot of key pluripotent genes TRA160 and SOX2 (left panel) in LoxP#10 (A, left panel) and RIPE#2 (B, left panel) hiPSCs. Bar plot quantification of percentage of LoxP#10 (A, middle panel) and RIPE#2 (B, middle panel) cells expressing both markers. Karyogram of LoxP#10 (A, right panel) and RIPE#2 (B, right panel) hPSCs. Figure S2: (A) Principal component analysis of pTriple hPSCs after 7 days of unbiased EB differentiation with upregulation of pancreatic master transcription factors.
