# Supplementary material for: A Recombinase-Mediated Cassette Exchange Platform for a Triple Independent Inducible Expression System for Human Pluripotent Stem Cells
Source: Cells. 2025 Jan 24;14(3):184. doi: 10.3390/cells14030184 (PMC11817695; doi:10.3390/cells14030184)
Supplement: Supplementary file 1 [file cells-14-00184-s001.zip › cells-3357686-supplementary.pdf]

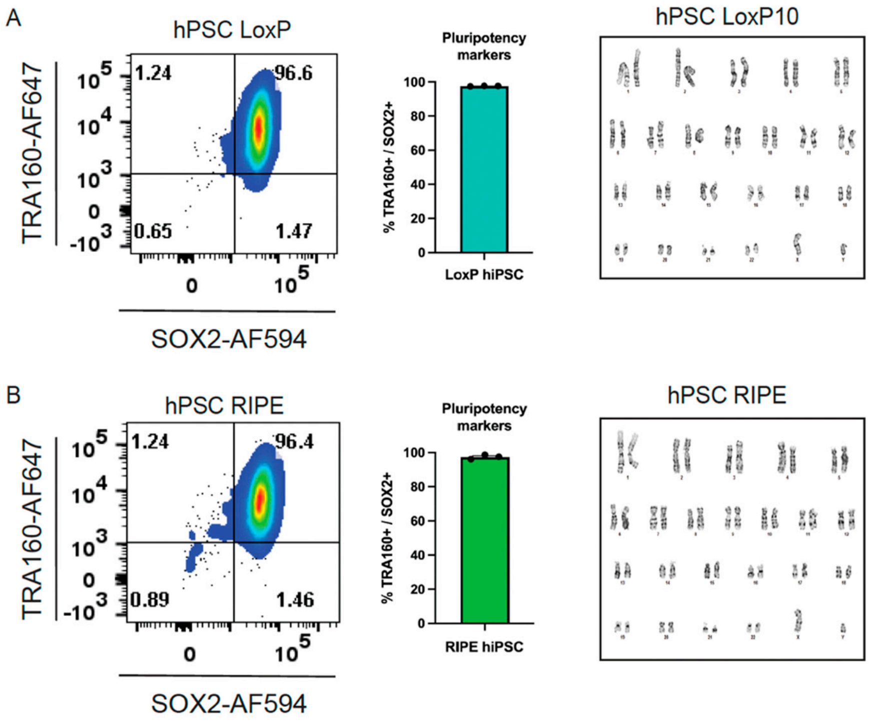

Supplementary Figure 1

Supplemental Figure S1. Characterization of LoxP#10 and RIPE#2 hiPSCs. Flow cytometric plot of key pluripotent genes TRA160 and SOX2 (left panel) in LoxP#10 (A, left panel) and RIPE#2 (B, left panel) hiPSCs. Bar plot quantification of percentage of LoxP#10 (A, middle panel) and RIPE#2 (B, middle panel) cells expressing both markers. Karyogram of LoxP#10 (A, right panel) and RIPE#2 (B, right panel) hPSCs.

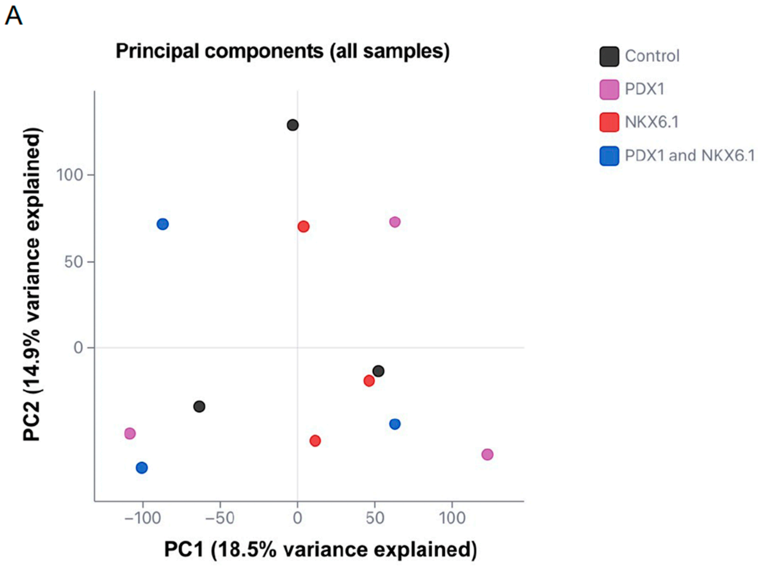

Supplementary Figure 2

Supplemental Figure S2. (A) Principal component analysis of pTriple hPSCs after 7 days of unbiased EB differentiation with upregulation of pancreatic master transcription factors.
